# Risk Factors and Preventive Measures for Breast Cancer

**DOI:** 10.3390/jcm13164610

**Published:** 2024-08-07

**Authors:** Marie-Christin Winkler, Svetlana Hetjens

**Affiliations:** Department of Medical Statistics and Biomathematics, Medical Faculty Mannheim, University of Heidelberg, Theodor-Kutzer-Ufer 1-3, D-68167 Mannheim, Germany; mc.franke@gmx.de

**Keywords:** prevention, breast cancer, mortality

## Abstract

**Background:** Breast cancer is the most common cancer in women in many countries. Breast cancer is a multifactorial disease. This study investigates the possible influencing factors and preventive measures for breast cancer. **Methods:** The data for this study were obtained from WHO databases. First, age standardization was performed, followed by a correlation analysis. Relationships between the mortality rates of breast cancer and the possible influencing factors were analyzed. The significant results from the correlation analysis were analyzed using a stepwise regression analysis. In order to find out whether the application of a uniform screening program can reduce breast cancer mortality, the countries were divided into groups according to the time of initiation of the screening program, and breast cancer mortality was compared. **Results:** The correlation analysis showed a significant relationship with breast cancer mortality for 15 of 30 potential influencing factors. A stepwise multivariate regression analysis was performed with these 15 factors, which revealed 13 relevant factors. Two factors were more relevant: the number of radiotherapy units per 100,000 inhabitants and the proportion of the population (over 50 years of age) with a social network. These were followed by the proportion of general practitioners and obstetricians, as well as healthcare expenses. Breast cancer mortality differed between the countries that introduced the screening program in the 1990s and those that did so in the 2020s. **Conclusions:** A country’s healthcare system influences breast cancer mortality through prevention, diagnosis. and treatment. Regular screening, counseling for socially disadvantaged women, and prompt treatment are important factors. In the future, prevention measures should also aim to strengthen the social environment. The prescription of social activities should be used as preventive care.

## 1. Introduction

In many countries, breast cancer is the most common cancer in women. Worldwide, the incidence of breast cancer was 2.3 million women in 2022, with a mortality of 670,000. Breast cancer mortality in high-income countries has fallen by 40% in recent decades [1]. In 2018, the incidence in more developed countries was 54.4 per 100,000 women, and the mortality rate was 11.6 per 100,000 women [2]. The opposite trend can be observed in less-developed countries: although the incidence is significantly lower at 31.3 per 100,000 women, due to poorer early detection, diagnosis, and treatment, the mortality rate of 14.9 per 100,000 women is significantly higher than in most developed countries [3]. There are also major regional differences. In the USA, breast cancer affects one in eight women; in Asia, only one in 35 women develop the disease [4].

Unlike most other cancers, breast cancer also affects young women. In most cases, however, middle-aged or older women are affected. The average age of onset is 63, but the risk of developing breast cancer increases steadily from the age of 45 [5].

The following types of breast cancer can be classified [6]:

- Ductal carcinoma in situ (DCIS): DCIS is a non-invasive breast cancer that begins in the milk ducts of the breast. It remains confined to the ducts and has not spread to the surrounding breast tissue. DCIS has a very good prognosis when detected and treated early.
- Lobular carcinoma in situ (LCIS): LCIS is also non-invasive and begins in the milk-producing glands. Unlike DCIS, LCIS does not spread, but it increases the risk of developing invasive breast cancer later. LCIS itself is not life-threatening, but it is a marker for an increased risk of breast cancer.
- Invasive ductal carcinoma (IDC): IDC begins in the milk ducts and invades the surrounding breast tissue. It can spread to other parts of the body. The prognosis depends on the stage and aggressiveness of the tumor.
- Invasive lobular carcinoma (ILC): ILC begins in the milk-producing glands and can spread to the surrounding breast tissue. It is the second most common form of invasive breast cancer after IDC. Similar to IDC, the prognosis depends on the stage and aggressiveness of the tumor.
- Inflammatory breast cancer: It is a rare but aggressive form of breast cancer that blocks the lymph vessels in the skin of the breast, leading to redness, swelling, and warmth. Due to its aggressiveness, the prognosis is often poorer than other types of breast cancer.
- Triple-negative breast cancer: This type of breast cancer is negative for estrogen receptors, progesterone receptors, and HER2. This means it does not respond to hormonal therapies or HER2-targeted therapies. Triple-negative breast cancer tends to be more aggressive and has a poorer prognosis than other types of breast cancer.
- HER2-positive breast cancer: This type of breast cancer has an overexpression of the HER2 gene, which drives tumor growth. It is an aggressive tumor.
- Estrogen receptor-positive (ER+) and progesterone receptor-positive (PR+) breast cancer: These types of breast cancers have receptors for the hormones estrogen and/or progesterone, which drive their growth. They tend to grow slowly and have a good prognosis.

The development of breast cancer is a multi-step process that affects many cell types [7]. Gene mutations greatly increase the risk of breast cancer, with mutations in the BRCA1 and BRCA2 genes being of particular note [1].

The most important proven risk factors for the development of breast cancer are [5,8]:

- Age;
- Family history of breast cancer;
- Situations with hormonal imbalance or hormone (replacement) therapy;
- Food composition, e.g., high-fat nutrition;
- Nicotine and alcohol consumption;
- Obesity and type II diabetes;
- Low physical activity;
- Radiotherapy of the chest during childhood (e.g., for lymphoma);
- Late first pregnancy (over 30 years of age) or childlessness;
- Less or no breastfeeding;
- The breast size;
- Early menstruation or late menopause.

The prevention strategy is made up of various aspects, including both lifestyle changes and medical interventions. Regular screening is the most important means of significantly improving the prognosis and minimizing mortality [7]. Mammography is the most widely used screening tool and has been shown to significantly reduce mortality [9]. In Europe, mammography screening saves around eight lives for every 1000 healthy women aged between 50 and 69 [10]. Normally, breast cancer can be treated surgically, with radiotherapy or chemotherapy, or with anti-hormonal or anti-proliferative therapy. In addition, bone-protective therapy can also improve the prognosis [11].

Environmental factors also play a role in the development of breast cancer. Studies of women from countries with a low incidence of breast cancer who emigrated to countries with a high incidence show that the risk of developing breast cancer adapted there within two generations [12]. Chemicals in the environment (bisphenol, phthalates, and parabens) can affect the hormone system and increase the risk of breast cancer [13]. Long-term exposure to certain pesticides and herbicides has been linked to an increased risk of breast cancer [14].

Many national programs have been developed, including both primary and secondary prevention measures, to reduce the incidence of and mortality from breast cancer. Even though initial success is already visible, too few women in Europe participate in breast cancer screening [15]. In addition, the quality of the screening programs offered greatly varies from country to country [16]. As mammography screening has been shown to reduce breast cancer mortality, this study analyzed compliance with and implementation of the European guidelines and possible effects on breast cancer mortality [9].

The aim of this study was to identify and demonstrate correlations by analyzing mortality data and different factors from European Union (EU) countries. The correlations shown could thus provide a better understanding of etiological factors and possible points of interventions to prevent breast cancer and ultimately provide an indication of how to reduce disease-specific incidence and mortality.

## 2. Materials and Methods

### 2.1. Screening Programs

In 2017, the European Commission published a report on the implementation of the guidelines. All European countries were asked whether the quality standards for breast cancer screening were met [17]. The introduction of a screening program was documented, and questions on funding (does health insurance cover the costs?) and follow-up (does the screening program actively invite people who test positive for further examinations?) were answered by all countries.

### 2.2. Mortality Rates

For this study, the mortality data from the “WHO mortality database” were selected for the following ICD-10 codes for breast cancer:

- C50: Malignant neoplasm of the mammary gland [mamma];
  Incl: Connective tissue of the mammary gland;
  Excl: Skin of the mammary gland (C43.5) (C44.5);
- C50.0: Acromastium and areola;
- C50.1: Central glandular body of the mammary gland;
- C50.2: Upper inner quadrant of the mammary gland;
- C50.3: Lower inner quadrant of the mammary gland;
- C50.4: Upper outer quadrant of the mammary gland;
- C50.5: Lower outer quadrant of the mammary gland;
- C50.6: Recessus axillaris of the mammary gland;
- C50.8: Mammary gland, several parts overlapping;
- C50.9: Mammary gland, unspecified.

The ICD-10 system is recognized worldwide. ICD stands for “International Statistical Classification of Diseases and Related Health Problems” [18]. Due to the different coding habits of the EU countries, the ICD codes listed above have been summarized.

### 2.3. Factors

In order to obtain a comprehensive picture of the factors influencing the etiology within the EU countries, this study not only analyzed several classic medical risk factors for breast cancer but also the circumstances and living conditions in the respective countries.

From the groups of country-specific, socio-economic, and health factors, potential influencing factors on the etiology of breast cancer were selected from the WHO database “European Health for all” (HFA).

Country-specific factors:

- Number of abortions per 1000 live births;
- Annual pure alcohol consumption per person in liters;
- Percentage of breastfed infants aged 3 months;
- Percentage of the prevalence of diabetes in the total population;
- Average amount of fruits and vegetables available per person per year (in kg);
- Percentage of tobacco smokers in the population (aged 15 and over);
- Proportion of obese people (defined as BMI = 25 kg/m^2^) in the total population;
- Breast cancer incidence per 100,000 inhabitants;
- Proportion of the population (over 50) with a social safety net;
- Proportion of all live births to mothers over 35 years of age;
- Proportion of all live births to mothers under 20 years of age.

Socio-economic factors:

- Gross national income per person (in USD);
- Total health expenses per person (in USD adjusted for purchasing power);
- Share of private sector health expenses in total expenses;
- Share of public sector health expenses in total expenses;
- Literacy rate in the population (aged 15 and over);
- Unemployment rate;
- Share of urban population in the total population.

Health determinants:

- Number of obstetricians and gynecologists per 100,000 inhabitants;
- Share of pharmaceutical expenses within healthcare expenses;
- Estimated life expectancy at birth;
- Mortality rate per 1000 inhabitants;
- Number of general practitioners per 100,000 inhabitants;
- Number of acute hospital beds per 100,000 inhabitants;
- Number of mammographers per 100,000 inhabitants;
- Number of all available mammographs;
- Number of MRI machines per 100,000 inhabitants;
- Number of positron emission tomography devices per 100,000 inhabitants;
- Number of irradiation devices per 100,000 inhabitants;
- Number of computed tomography devices per 100,000 inhabitants.

### 2.4. Statistical Methods

In order to find out whether the application of a uniform screening program can reduce mortality from breast cancer, the countries were divided into different groups according to the time of initiation of the screening program. The mean mortality rate was calculated and presented as a mean value with standard deviation (SD). An analysis of variance (ANOVA) was then used to check whether the countries that introduced screening early differed in breast cancer mortality from the countries that introduced screening late or not at all. A Scheffé adjustment was performed to adjust the alpha level for multiple testing. The questions on funding and follow-up were analyzed using a *t*-test. To analyze the mortality data and possible influencing factors, the data for the period from 2010 to 2015 were analyzed. Direct age standardization of the mortality data was carried out in order to rule out the possibility that mortality differences between these countries were due to different age structures. Germany was used as the standard population. The following classes (k) were used: k1 = 15–40, k2 = 40–60, and k3 = over 60. As children and adolescents were not considered in this study, the first class, k1, started at the age of 15. Only the data from women were analyzed, due to their relevance and also for better statistical significance. The Pearson correlation analysis was performed to analyze the relationships between the selected quantitative influencing variables and the mortality rates. A multiple stepwise regression analysis was then carried out to analyze several influencing variables simultaneously. The stepwise regression analysis provided a test variable (F), according to which the *p*-value was calculated. The larger this value was, the more important the parameter was in the model, and therefore, it was also the most important factor with the most influence on the dependent parameter (mortality rate). Multiple imputation (a missing data method) was used for this analysis. The *p*-value of <0.05 was considered significant as the statistical significance level. The analysis was performed using SAS 9.4 software.

## 3. Results

### 3.1. Analysis of the Screening Programs

**Figure 1:**
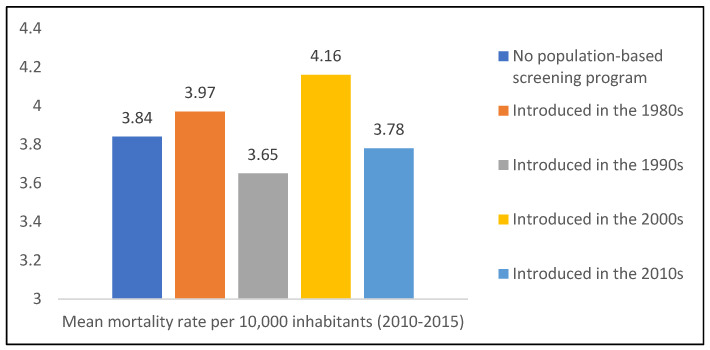
Introduction of breast cancer screening. *No population-based screening program*: Bulgaria, Greece, and Slovakia. *Introduced in the 1980s*: Finland 1987, The Netherlands 1989, Sweden 1986, and United Kingdom 1988. *Introduced in the 1990s*: Italy 1990, Luxembourg 1992, Portugal 1990, and Spain 1990. *Introduced in the 2000s*: Belgium 2001, Croatia 2006, Cyprus 2003, Czech Republic 2002, Denmark 2008, Estonia 2003, France 2004, Germany 2005, Hungary 2001, Ireland 2000, Latvia 2009, Lithuania 2005, Malta 2009, Poland 2006, and Slovenia 2008. *Introduced in the 2010s*: Austria 2014 and Romania 2015.

From this 2017 European Commission report, the following groups were formed based on the decade of the introduction of breast cancer screening (Figure 1).

The breast cancer mortality rate was only significantly different between screenings introduced in the 1990s and in the 2000s (*p*-value: 0.0004).

The *t*-test comparing the two groups with and without cost sharing by the health insurance company showed no significant difference between the two groups, with a *p*-value of 0.1169. The following groups were formed:

- Group A: Full or partial participation of health insurance. The mean mortality rate was 4.12 (SD: 0.57).
- Group B: No co-financing. The mean mortality rate was 3.96 (SD: 0.55).

A further *t*-test was carried out to determine whether the active invitation to further examinations in the event of a positive result had an effect on breast cancer mortality. The following groups were formed:

- Group C: Women with abnormal findings in the screening were actively invited for further examinations. The mean mortality rate was 3.98 (SD: 0.58).
- Group D: the women with abnormal findings were not actively invited for further examinations. The mean mortality rate was 3.99 (SD: 0.50).

Again, there was no significant difference between the groups, with a *p*-value of *p* = 0.6254.

### 3.2. Analysis of the Healthcare System

The healthcare system was analyzed by the following factors in the correlation analysis:

- Share of pharmaceutical expenses within healthcare expenses (*p* = 0.0017);
- Number of general practitioners per 100,000 inhabitants (*p* = 0.0092);
- Number of acute hospital beds per 100,000 inhabitants (*p* = 0.9311);
- Number of mammographers per 100,000 inhabitants (*p* = 0.5877);
- Number of all available mammographs (*p* = 0.0817);
- Number of MRT machines per 100,000 inhabitants (*p* = 0.2527);
- Number of positron emission tomography devices per 100,000 inhabitants (*p* < 0.0001);
- Number of irradiation devices per 100,000 inhabitants (*p* = 0.0001);
- Number of computed tomography devices per 100,000 inhabitants (*p* = 0.7465).

### 3.3. Analysis of the Mortality Data and the Possible Influencing Factors

A correlation analysis was carried out between all selected potential influencing factors and the mortality rate. Here, 15 out of 30 potential influencing factors showed a significant correlation with breast cancer mortality. A stepwise multivariate regression analysis was then carried out with these 15 significant factors from the correlation analysis. The 13 factors that were found to be relevant are shown in Table 1 and were sorted according to the F-test size.

The most important factor was the number of radiation systems per 100,000 inhabitants, followed by the proportion of the population (over 50 years) with a social network.

## 4. Discussion

Numerous studies have already investigated various possible factors influencing the risk of developing breast cancer. For some of these factors, a clear link to the development of breast cancer has been established, while for other factors, there are conflicting results, or no link has been established. Some of the important influencing factors, such as unhealthy nutrition, obesity, alcohol consumption, low physical activity, age at first pregnancy, breastfeeding, and hormone replacement therapy, differ between European countries [19]. Other important risk factors that differ between countries are differences in genetic predisposition and the availability and access to early detection, diagnosis, and treatment of breast cancer [20].

In this study, the countries of the EU were analyzed with regard to breast cancer mortality. Determinants and possible influencing factors from country-specific, socio-economic, and health factors were taken into account and placed in the context of breast cancer mortality. In addition, compliance with and implementation of the European guidelines and possible effects on breast cancer mortality were also analyzed.

The data for the comparison of the implementations of the guidelines were analyzed from the European Commission’s 2017 report. The countries were divided into five groups. Group 1 contained the countries in which there was no screening program at all. In the countries in group 2, screening was already introduced in the 1980s; in group 3, screening was introduced in the 1990s; in group 4, screening was introduced in the 2000s; and in group 5, screening was introduced in the 2010s. There was a significant difference between groups 3 and 4: mortality was lower in group 3, where a screening program was introduced, on average, ten years earlier than in group 4. A meta-analysis of 60 studies also showed that organized screening reduces breast cancer mortality [21]. In group 2, the screening program was introduced another 10 years earlier, i.e., in the 1980s. However, there was no significant difference between group 2 and the other groups (with later introduction of the screening program). One possible explanation for the result would be that the benefit of screening in the 1980s was not yet so high, due to the poorer quality of the equipment and less experienced examiners (radiologists), which could have had a positive effect on the mortality rate.

Two *t*-tests were then carried out to find out how funding (does health insurance cover the costs?) and follow-up (does the screening program actively invite those who test positive for further examinations?) affected the breast cancer rate. The results were not statistically significant, indicating that neither cost-sharing by health insurance companies nor an active invitation of positively screened patients for further examinations has a relevant effect on breast cancer mortality. The influence of these factors is probably too small, but they should continue to be surveyed as part of EU-wide quality assurance.

The data from the WHO databases were used to analyze the correlations between the mortality rate and the possible influencing factors. The period of six years was limited, so only trends in the direction of development were described. In addition, the following limitations applied: The missing values in the regression analysis were supplemented using multiple imputation, as this was the only way to analyze all countries and factors. However, multiple imputation is only an estimation. It remains unclear whether the actual values would have delivered the same results.

Physical activity and a healthy nutrition can help reduce the risk of breast cancer [15]. The risk of developing breast cancer can be reduced by 10–25% through physical activity [22]. In this study, there was no suitable influencing factor with sufficient data to represent the physical activity of the population in the respective country. For future studies, it would be useful to analyze the physical activity habits in the respective countries. Healthy nutrition was depicted with the factor “available amount of fruit and vegetables per year in kg”. However, no significant correlation was found in the correlation analysis. This could be due to the time distance between a healthy diet and positive effects on cancer risk. For future studies, it would be important to obtain even better evidence for the individual nourishing factors and the combination of different factors and to observe patients and their nutritional habits in the long term.

The consumption of high-calorie foods, sugary foods, and alcoholic beverages should be avoided [23,24]. Weight increase up to the obesity level (BMI > 30) is associated with a high risk of breast cancer [25]. The factor “proportion of obese people in the total population” was examined in this context and found to be insignificant. Two factors correlate if they have the same increase, but if there are already many obese individuals in the population (i.e., this factor remains roughly the same) and the mortality rate increases, then there is no significant correlation. The risk of developing cancer increases with the dose of alcohol, and the metabolic product acetaldehyde is responsible for this [11]. The factor “annual pure alcohol consumption per person in liters” is not significant, but data from the WHO database include both sexes. This could lead to a distortion of the results, as breast cancer mortality was only considered for women. Knight et al. were also able to prove in the WECARE study that the risk of breast cancer is increased in women who both drink and smoke [26].

Age at first pregnancy and breastfeeding are important factors. A young age at first pregnancy and any subsequent births before the mother’s 30th birthday have a protective effect on the development of breast cancer [27]. Women who do not breastfeed have a higher risk of developing breast cancer than women who breastfeed [11]. Prolonged breastfeeding reduces the influence of estrogen and progesterone on breast tissue, thereby reducing the risk of breast cancer [12]. There were too little data available to analyze these factors; all ages at pregnancy and the exact duration of breastfeeding would be needed.

Some studies and meta-analyses show a link between the long-term use of oral contraceptives and the risk of developing breast cancer [28,29,30]. However, the fact that women who take the contraceptive pill have children later overall could falsify the result. In addition, the use of oral contraceptives is associated with a significant reduction in the risks of ovarian cancer, endometrial cancer, and colorectal cancer. Calculations assume that taking oral contraceptives for at least five years shows a slight reduction in the overall risk of developing cancer [29,31]. Due to the sometimes contradictory results from past studies, prospective randomized clinical studies on the influence of pill use on the risk of breast cancer should be carried out, especially with modern preparations that contain lower doses of hormones.

Mortality from breast cancer and other diseases also depends on the socio-economic status of a country. For example, it has been shown that women from socioeconomically disadvantaged regions of Europe have a lower incidence of breast cancer but a higher mortality rate from breast cancer [32]. Higher public healthcare expenses are associated with a reduction in breast cancer mortality [33]. Public healthcare expenses and private expenses are also significant in this study. Income and insurance status are also associated with breast cancer [34]. A country’s healthcare system influences the prevention, diagnosis, treatment, progression, and mortality of breast cancer. The analysis of the healthcare systems revealed three significant factors: share of pharmaceutical expenses within healthcare expenses, number of positron emission tomography devices per 100,000 inhabitants, and number of irradiation devices per 100,000 inhabitants. The factor “share of pharmaceutical expenditure in healthcare expenditure” was the only one of the three factors that remained in the stepwise multiple regression. Vrdoljak et al. found that the biggest difference between western Europe and central and eastern Europe lies in spending on cancer treatments. In their cross-sectional study, they examined the correlation between the mortality-to-incidence rate and the expenditure on tumor drugs between western Europe and central and eastern Europe. They found that there are large differences between the expenditures on tumor drugs [35]. Higher spending on tumor drugs can therefore reduce mortality, and the differences between EU countries could be decreased. The most important factor in breast cancer mortality in this study turned out to be the “number of radiation units per 100,000 inhabitants”. A high number of radiation units and a high incidence could reflect a need for treatment places. However, the sheer number of radiation units available says nothing about their quality and therefore the success of the treatment. The second most important factor was “proportion of the population (over 50) with a social network”. The strengthening of the social environment can be promoted by social prescribing. Social prescribing allows physicians to prescribe social and community activities [36,37]. This would expand the social network and potentially reduce breast cancer mortality. In addition, initial results of social prescribing interventions indicate lower healthcare expenses [38].

## 5. Conclusions

A country’s healthcare system influences breast cancer mortality through prevention, diagnosis, and treatment. Regular screening, counseling for socially disadvantaged women, and prompt treatment are important factors in this regard. In the future, prevention measures should also aim to strengthen the social environment. The prescription of social activities should be used as a preventive care.

## Figures and Tables

**Table 1:** Stepwise multiple regression for breast cancer in women; R^2^ = 0.76.

Parameter	*p*-Value	F-Value
Number of radiation systems per 100,000 inhabitants	<0.0001	172.09
Proportion of the population (over 50) with a social network	<0.0001	107.08
Number of general practitioners per 100,000 inhabitants	<0.0001	106.34
Number of obstetricians and gynecologists per 100,000 inhabitants	<0.0001	76.99
Total healthcare expenses per person	<0.0001	65.61
Proportion of all live births to mothers under 20 years of age	<0.0001	59.92
Amount of private sector healthcare expenses in total expenses	<0.0001	58.16
Amount of public sector healthcare expenses in total expenses	<0.0001	53.23
Number of positron emission tomography systems per 100,000 inhabitants	<0.0001	50.45
Amount of pharmaceutical expenses in healthcare expenses	<0.0001	48.45
Unemployment rate	<0.0001	37.61
Breast cancer incidence per 100,000 inhabitants	<0.0001	28.92
Gross national income per person (in USD)	<0.0001	15.67

## Data Availability

The data in this study came from WHO databases. The data presented in this study are available upon request from the corresponding author.
